# A Close Look at Echium amoenum Processing, Neuroactive Components, and Effects on Neuropsychiatric Disorders

**DOI:** 10.31661/gmj.v8i0.1559

**Published:** 2019-10-11

**Authors:** Mohammad Nouri, Fereshteh Farajdokht, Mohammadali Torbati, Fatemeh Ranjbar, Sanaz Hamedyazdan, Mostafa Araj-khodaei, Saeed Sadigh-Eteghad

**Affiliations:** ^1^Neurosciences Research Center, Tabriz University of Medical Sciences, Tabriz, Iran; ^2^Department of Persian Medicine, School of Traditional Medicine, Tabriz University of Medical Sciences, Tabriz, Iran; ^3^School of Nutrition and Food Sciences, Tabriz University of Medical Sciences, Tabriz, Iran; ^4^Psychiatry Research Center, Tabriz University of Medical Sciences, Tabriz, Iran; ^5^Drug Applied Research Center, Tabriz University of Medical Sciences, Tabriz, Iran; ^6^Aging Research Institute, Tabriz University of Medical Science, Tabriz, Iran

**Keywords:** *Echium amoenum*, Rosmarinic Acid, Phenolic Compound, Antioxidant, Anti-inflammatory, Neuropsychiatric Disorders, Persian Medicine

## Abstract

Pharmacological researches in the area of herbal medicine have considerably increased over the last two centuries. *Echium amoenum* (known as *Gol-e-Gavzaban* in Persian) is a medicinal plant that has been widely used in Iranian folk medicine. In this review, databases including PubMed, Scopus, and Google Scholar were searched up. Data collecting was completed by January 2019 and available scientific reports regarding the processing methods, main chemical constituents, and effects of *E. amoenum* on different neuropsychiatric disorders are summarized. Thirteen five studies met the inclusion criteria. According to results, the important phytochemicals of the plant was phenolic compounds, fatty acids, rosmarinic acid, anthocyanidins, and flavonoids. Also, experimental and clinical studies demonstrated the effectiveness of *E. amoenum* in the treatment of several neuropsychiatric disorders such as anxiety, depression, ischemic stroke, seizure, Alzheimer’s disease, and pain. Many of these effects are, at least in part, due to its rosmarinic acid or polyphenolic compounds such as flavonoids and natural pigments such as anthocyanins. Also, fatty acids such as gamma-linolenic acid play critical role in neuroactive properties of this herb. Among these effects, only the antidepressant and anxiolytic properties of the plant extract have been examined both experimentally and clinically. There was some controversy over its toxicity effects. It seems that *E. amoenum* protects neurons via attenuation of oxidative stress and inflammation as well as blocking of apoptosis in the nervous system. However, more studies are necessary for assessing exact mechanisms of action in neuropsychiatric disorders, finding of bioactive ingredients, and processing methods of this plant.

## Introduction

**Figure 1:**
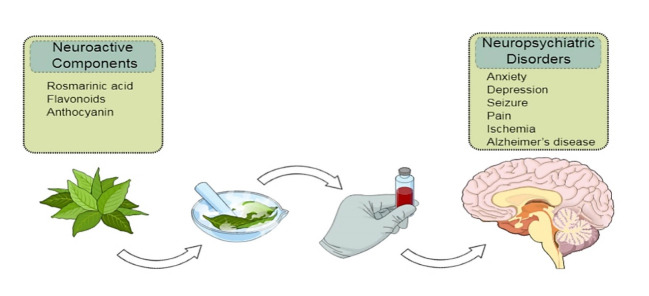
Schematic shows neuroactive components of *Echium amoenum* and their effects on different neuropsychiatric disorders.

Over the past decade, the use of medicinal plant products gain more attention compared to synthetic chemical drugs possibly due to their lighter side effects [1]. According to the World Health Organization at least 80% of people worldwide, particularly in developing countries, trust herbal medicine for primary healthcare [2, 3]. *Echium amoenum* Fisch & Mey, belonging to *Boraginaceae* family, is a famous biennial Iranian medicinal plant which is naturally grown North of Iran, and also in Europe and Mediterranean regions [4]. The *E. amoenum* is different in genus and species from the borage grown in Europe, *Borago officinalis* L. (Boraginaceae). The *E. amoenum* has russet-red and funnel-shaped flowers that after drying can be seen in violet-blue, while *B. officinalis* has bright-blue and star-shaped flowers [5]. The main medicinal parts of the *E. amoenum* plant are flowers and the hairy lance-shaped leaves which have been used for therapeutic purposes in different parts of the world [6, 7]. Phytochemical research showed that *E. amoenum* contains different bioactive compounds such as rosmarinic acid (RA), anthocyanidins, flavonoids, the trace of alkaloids, saponins, unsaturated terpenoids, and sterols [8, 9]. The main essential oils of *E. amoenum* flowers are palmitic acid n-tricosane, linoleic acid, n-pentacosane, and pulegone [10]. Moreover, the main fatty acids of *E. amoenum* seeds are linoleic acid, linoleic acid, oleic acid, palmitic acid, gadoleic acid, linoleic acid, and stearic acid [10]. Nutritional compositions of *E. amoenum* (Figure-1) include phosphorous, calcium, iron, vitamin C, beta-carotene, niacin, riboflavin, thiamine, and silicic acid [11]. Its bioactive components are used as a novel source of materials in food, drug, and cosmetic industries.The advantages of this plant have initially been found by the Romans 300 B.C [12]. The sedative and mood enhancer effects of this plant have been reported in Persian medicine (PM) manuscripts, for instance, the *Tohfat-Al-Hakim, Makhzanoladvieh*, and *Canon of medicine* [13, 14]. It is widely used for subsiding common cold symptoms such as sore throat, coughing, and pneumonia in PM. Anti-inflammatory, antibacterial, antioxidant, analgesic, anxiolytic, sedative, antidepressant, and immunomodulatory effects of this plant have been reported in recent studies [15]. Beneficial effects of aqueous extract of this plant in the management of the obsessive-compulsive disorder, anxiety, depression, seizure, pain, brain ischemia, and Alzheimer’s disease (AD) have also been reported [16-21]. The aqueous extract of *E. amoenum* petals has also been reported to reduce blood lipid peroxidation levels and increase total antioxidant capacity (TAC) and total thiol in human, suggesting its potential antioxidant activity [22]. Moreover, *E. amoenum* extract is capable of diminishing inducible NO synthase (iNOS), and cyclooxygenase-2 (COX-2) enzymes activities, and decrease pro-inflammatory cytokines levels such as tumor necrosis factor (TNF)-α, interleukin (IL)-1β, and IL-6 [23]. Despite the extensive use of *E. amoenum*, there is no comprehensive review regarding its neurological activities and the chemical constituents. In this review, we summarized the applications of *E. amoenum* in the treatment of different neuropsychiatric disorders and its possible mechanisms of action. The results of this review may provide strong evidence for the effectiveness of *E. amoenum* related products in the neurosciences.

## Search Strategies

Multiple databases including PubMed, Scopus, and Google Scholar were searched up with the exact keyword “*Echium amoenum*” to get recent researches relevant to the use of *E. amoenum* in the treatment of neuropsychiatric disorders. Data collecting was completed by January 2019. Search keywords were “*Echium amoenum*” AND “cognition” OR “brain function” OR “brain structure” OR “oxidative stress” OR “antioxidant” OR “psychiatry” OR “psychology” OR “depression” OR “anxiety” OR “Alzheimer” OR “Alzheimer’s disease” OR “stroke” OR “ischemia” OR “neuroinflammation” OR “toxicity” were the keywords used in the search process. The papers focusing on the effect of this plant on the nervous system as well as the phytochemical and pharmacological properties of *E. amoenum* were collected. However, studies related to agriculture and genetics were excluded.

## Results

In the first step, 186 studies were found. After applying the exclusion criteria, 35 studies were eligible for inclusion. Some papers investigated phytochemicals properties and chemical constituents of the plant, such as phenolic compounds, fatty acid profile, RA, anthocyanidins, and flavonoids contents. The yielded papers reported the pharmacological effectiveness of *E. amoenum* in neurological disorders, including anxiety, depression, pain, stroke, AD, and seizure. Among these effects, only the antidepressant and anxiolytic properties of the plant extract have been examined both experimentally and clinically. There was some controversy over its toxicity effect, which are discussed in this review.

## Discussion

### 1. Effect of Environmental Factors and Processing Methods On Bioactivity of E. amoenum

#### 1.1. Environmental Factors

Several factors such as climate, harvesting time, geographical location, as well as parts of the plant, and methods of drying or extraction can affect the constituents of *E. amoenum* [10, 24]. Interestingly, evidence shows that extracts from different organs of *E. amoenum* such as stem, seeds, or petals have different amounts of phenolic compounds and antioxidant activity. Moreover, geographical locations affect the bioactivity of *E. amoenum*. For instance, seed and stem extracts of *E. amoenum* from Northern cities of Iran with humid and cold weather, have the highest antioxidant activity and total phenolic contents [25]. Another study also reported that the content of anthocyanins and flavonoids of *E. amoenum* are augmented with increased altitude and decreased temperature [26]. Moreover, soil salinity could affect ion concentrations of flowers, leaves, and stem of *E. amoenum* so that a high concentration of salt in soil decreases K and Ca contents while increases Na content [27].

#### 1.2. Drying

To ensure the quality of medicinal plants and their products, the proper parts of the medicinal plant must be harvested during the optimal stage of development. Drying is a standard way for conservation of medicinal plants after harvest, which facilitates their handling and storage [28]. Drying conditions, particularly drying temperature and air velocity can affect the final quality of the product, and the ideal way for drying of *E. amoenum* petals is shade drying [29]. However, sun drying is not recommended for medicinal plants because it can lead to photodecomposition reactions [30]. It has been shown that drying decreases antioxidant capacity, total phenolic, and total flavonoid and anthocyanin contents of fresh *E. amoenum* petals possibly due to thermal degradation and a decrease in moisture content. The highest content of bioactive compounds for this herb is obtained after drying at high temperature (60°C) and intermediate air velocity (0.86 m/s) [31]. Therefore, the drying of this plant must be carefully performed to preserve its bioactive components.

#### 1.3. Extraction Methods

The extraction method and type of solvents are also important factors which can affect the phytochemical contents and biological activities of extracts of *E. amoenum* plant. In this regard, an *in vitro* study investigated antioxidant activities of different extracts from *E. amoenum* petals including ultrasonically assisted extraction (extracted with methanol in an ultrasonic bath), alkaloid rich (extracted with methanol by infusion), and polyphenol (extracted with methanol/acetone/water) extracts. Their results showed that polyphenol fraction had the highest and alkaloidal fraction had the lowest total phenolic contents. Moreover, the ultrasonic extract had the highest and polyphenol had the lowest flavonoid contents. Furthermore, the ultrasonic extract showed the highest and polyphenol extract showed the lowest 1,1-diphenyl-2-picrylhydrazyl (DPPH) radical-scavenging activity. Moreover, polyphenol extract had the highest scavenging of H_2_O_2_ activity as well as Fe^+2^ chelating ability [32]. A recent study examined the effect of various extraction methods of *E. amoenum* petals on phenolic content, RA content, antioxidant capability, and ability to inhibit several enzymes activities. The results showed that the methanolic, decoction, and infusion extracts of *E. amoenum* contained high phenolic content. Furthermore, the decoction (prepared at 100 °C) has the highest ability to extract phenolic compounds, because *E. amoenum* polyphenols have the great solubility in hot water [11]. The determination of the amount of RA in the extracts also showed that the infusion, decoction, and methanolic extract, respectively had high RA content, indicating that water and temperature are compelling factors affecting the extraction of RA. Moreover, decoction had the uppermost total antioxidant activity and radical scavenging capacity among other extracts and its half-maximal inhibitory concentration (IC50) value was higher than the standard antioxidant (ascorbic acid). Additionally, the decoction had the highest tyrosinase inhibitory action while methanolic extract demonstrated superior cholinesterase inhibitory activity [33]. Previous studies have proven high solubility of phenolic compounds of *E. amoenum* in water, and these three methods are powerful in the extraction of phenolic contents and antioxidant substances. Moreover, the strongest antioxidant activity in this extract is obtained with higher polarity solvents. Investigation of the antioxidant activity of different extracts of *E. amoenum* , including water, acetone, ethanol, 80% ethanol, methanol, and 80% methanolic extracts have also revealed that the water extract at 100°C and at 30°C had the highest while acetone extract had the lowest antioxidant activity [11].

### 2. Main Chemical Constituents

#### 2.1. RA

The RA, caffeic acid ester of 3-(3,4-dihydroxyphenyl) lactic acid, often presents in species of the Boraginaceae, Lamiaceae, and Marantaceae families. The phytochemical studies reported that RA is the main phenolic compound of the ethyl acetate extract of *E. amoenum* petals [6]. The HPLC analysis revealed that the total RA content of *E. amoenum* extract ranged from 2.02% to 2.40% [33]. The RA has been broadly applied in traditional medicines in the management of several conditions including inflammation, infections, weakness and fatigue, and memory enhancement [34]. The medicinal value of RA is related to its anti-viral, antibacterial, analgesic, anxiolytic, antioxidant, anti-inflammatory, anticholinesterase, and neuroprotective activities [35-39]. Some in vitro and in vivo studies have proven anti-inflammatory and immunological effects of RA. It seems that its anti-inflammatory effect is via modulation of the complement pathway and inhibition of prostaglandins formation at the site of inflammation [40, 41]. Studies have also demonstrated that RA has a robust antioxidant activity even greater than vitamin E [35, 42]. In one study, the protective effect of RA against hydrogen peroxide (H2O2)-induced apoptosis has been shown in human dopaminergic neuronal cells through attenuation of reactive oxygen species (ROS), increasing the expression of *Bcl-2* (an anti-apoptotic protein), decreasing the expression of *BAX* (a pro-apoptotic protein), as well as an enhancement in the activity of heme oxygenase-1 (an antioxidant enzyme) [43]. Additionally, it has been found that RA attenuates the degeneration of motor neurons and improves motor performance in a mouse model of amyotrophic lateral sclerosis [44]. This agent can also improve spatial memory performance, which is attributed to the inhibition of prolyl oligopeptidase in rodents [45]. Several in vitro and in vivo reports also showed that RA could attenuate AD-associated amyloid-beta (Aβ) neurotoxicity and memory impairment [46, 47]. Furthermore, its inhibitory effects on acetylcholinesterase (AChE) activity in the rat’s brain have been approved [48].

#### 2.2. Flavonoids

Another constituent of *E. amoenum* are flavonoids, whose total content ranged from 4.14 to 11.11 mg QE/g of the extract [32]. Flavonoids are natural compounds with polyphenolic structure and low molecular weight which are found in fruits, vegetables, cereals, and teas. According to their molecular structures, they are categorized into six main groups including flavanols, flavanones, flavonol, isoflavones, anthocyanins, and catechins [49]. Flavonoids have several health benefits including antioxidant, anti-inflammatory, antiatherosclerotic, anticancer, antiosteoporotic, antiviral, antibacterial, anti-platelet aggregation, anti-ischemic, and vasodilatory properties [49-51]. Moreover, they can suppress ROS formation and enhance antioxidant defense activity [22, 52-54]. Several molecular mechanisms have been suggested for the anti-inflammatory activities of flavonoids. Inhibition of pro-inflammatory mediators (C-reactive protein) and cytokines (TNF-α, IL-1β, IL-6, IL-8) secretion, as well as the blockade of enzymatic activity of COX-2, lipoxygenase, and iNOS are some of the proposed mechanisms. Also, anti-inflammatory effects of flavonoids are attributed to the inhibition of nucleic factor-kappa b (NF-kB) and activating protein-1(AP-1) and enhancement the release of IL-10 [55]. In light of these activities, flavonoids are regarded as multi-target compounds that can be useful in the management of several neurological diseases comprising AD, Parkinson’s disease (PD), depression, epilepsy, and schizophrenia [56, 57]. A recent in vitro study has reported that natural flavonoid protects against Aβ-induced cytotoxicity through inhibiting the accumulation of ROS [58]. Inhibitory effect of flavonoids on AChE activity has also been reported [59]. Previous studies have also shown that chronic treatment with extracts enriched flavonoids enhances spatial working memory in mice possibly by increasing brain trophic factors, namely brain-derived neurotrophic factor (BDNF) [60, 61]. Besides, flavonoid-rich extracts have anticonvulsant activity in animal models of epilepsy [62, 63].

#### 2.3. Anthocyanin

Anthocyanins, natural pigments pertaining to the flavonoid family, possess antioxidant anti-inflammatory, anticarcinogenic, and cardioprotective properties [64]. Anthocyanins, especially cyanidin 3-O-glucoside (C3G), protect against traumatic spinal cord injury and improve functional recovery, decrease lesion volume and motor neuron damage via reducing the production of ROS in the injured site [65]. An in vitro study found that anthocyanins-rich extract reduced Aβ-induced neurotoxicity and apoptosis in PC12 cells through attenuation of ROS levels and caspase-3 activity, and improvement of mitochondrial membrane potential [66]. Moreover, anthocyanins can inhibit inflammatory response through inhibition of iNOS and COX-2 activities and attenuation of microglia activation [67]. The anthocyanins also protect cultured cerebellar granule neurons against mitochondrial oxidative stress-induced apoptosis via inhibition of lipid peroxidation and enhancement of mitochondrial glutathione (GSH) activity [68]. In a PD in vitro model, anthocyanins-rich extract alleviated rotenone-induced dopaminergic cell death through the improvement of mitochondrial function [69]. Moreover, it has been reported that anthocyanins protect the endothelium and improve capillary perfusion [70]. In vivo studies also showed that anthocyanins could easily cross the blood-brain barrier and improve memory function in aged mice model mainly through their antioxidant and anti-inflammatory activities [61, 67, 71]. Another study reported that pretreatment with anthocyanins prevents scopolamine-induced memory impairment, decreases AChE activity in the cortex and hippocampus, and restores hippocampal Na^+^, K^+^-ATPase, and Ca^2+^-ATPase activities [72]. Furthermore, pretreatment of anthocyanins can protect the brain against focal cerebral ischemia-induced cell damage through inhibition of phospho-c-Jun N-terminal kinase (p-JNK) and p53 signaling pathways [73]. The most common anthocyanin (13%) in the *E. amoenum* petals is C3G, which has a neuroprotective effect partly through attenuation of brain superoxide levels and blocking the release of apoptosis-inducing factors from mitochondria [74]. Delphinidin is another anthocyanin of *E. amoenum,* which decreases TNF-induced COX-2 expression [75, 76]. In an in vitro study, pretreatment with anthocyanin-rich extract of *E. amoenum* petals at concentrations of 25-1000 μg/mL has been shown to reduce H_2_O_2_-induced oxidative stress and increased ferric reducing antioxidant power in human endothelial cell culture [77].

#### 2.4. Gamma Linoleic Acid

The main identified fatty acids in *E. amoenum* seed include linolenic acid (35.69%-45.27%), linoleic acid (20.68%), and oleic acid (17.08%). Gamma-Linolenic acid (GLA)– a n-6 polyunsaturated fatty acid– presents in several plant seed oils such as borage, *E. amoenum*, black currant, hemp, as well as evening primrose oil. GLA possesses impressive protective effects against chronic inflammation, dermatitis, rheumatoid arthritis, diabetes, neuropathy, cardiovascular disease, obesity, atherosclerosis, and cancer [78]. GLA plays an essential role in modulating inflammatory responses. GLA competes with arachidonic acid inhibiting its conversion to detrimental inflammatory molecules by COX-2 and lipoxygenase enzymes. Moreover, GLA inhibits the activation of NF-ϰB signaling and activates peroxisome proliferator-activated receptor (PPAR) system [79, 80]. Previous studies have also shown that linoleic acid and GLA are safe in epilepsy, and their prolonged oral administration has a protective effect against seizures in rats [81]. A human study reported that 6-month prophylactic administration of GLA in migraineurs reduced the severity, frequency, and duration of the migraine attack, as well as decreased nausea and vomiting [82]. Moreover, GLA has been shown to improve diabetic neuropathy both in human and experimental diabetes model [83, 84].

### 3. Effect of E. amoenum on Neuropsychiatric Disorders

#### 3.1. Anxiety

The characteristic features of anxiety disorders range from behavioral patterns to changes in physiology including excessive fear, panic attack, uneasiness, *shortness of breath*, heart palpitations, dry mouth, sweating, and sleep disturbances [85]. Evidence shows that anxiety is associated with stimulation of the autonomic nervous system, especially the sympathetic nervous system, and dysregulation of the hypothalamus–pituitary–adrenal axis [86]. Treatments of anxiety disorders include psychological therapy, pharmacotherapy, or a combination of both [87]. One of the hot topics in psychopharmacology research is the application of traditional remedies as an appropriate treatment for mood disorders such as anxiety and depression. Evidence shows that many patients with psychiatric diseases prefer herbal medicines to chemical drugs for the treatment of their diseases [88]. The anxiolytic effect of the *E. amoenum* has been shown in acute and chronic administrations in rodents. Intraperitoneal administration of different dosage (62.5, 125, 250, and 500 mg/kg) of the aqueous extract of *E. amoenum* flower, 30 min before the test, exerted a mild to moderate anxiolytic effect in mice, and 125 mg/kg was the optimal dosage for anxiolytic effect [89]. The anxiolytic effect of ethanolic extract of *E. amoenum* flowers (50 mg/kg, i.p) has also been shown in the elevated plus maze (EPM) accompanied by decreased spontaneous locomotor activity indicating a sedative effect. Moreover, *E. amoenum* decreased ketamine-induced latency to sleep but did not affect the total duration of the sleep in rats [90]. In another study, administering aqueous extract of *E. amoenum* (125 mg/kg, i.p) during two different treatment courses (15 or 30 days) showed that *E. amoenum* induces a time-dependent anxiolytic effect in the EPM [91]. Moreover, Rabbani *et al*. demonstrated anxiolytic effects of hydroalcoholic extract of *E. amoenum* at the doses of 25 and 50 mg/kg after acute (30 min prior the tests) and chronic (for seven consecutive days) administrations in the light/dark box and EPM in mice. Interestingly, they reported that the anxiolytic effects of *E. amoenum* did not reduce after seven days of treatment indicating the lack of tolerance development [92]. Since tolerance to anxiolytic effects of synthetic drugs limits their long-term use, herbal remedies such as *E. amoenum* may provide a promising benefit in the treatment of anxiety disorders over long-term treatment. In another study, the anxiolytic effect of the herbal tea of *E. amoenum* was compared in the male and female rats. The results indicated that administration of *E. amoenum* ad libitum 24 h before the behavioral tests, in female rats induced anxiolytic effects stronger than male rats, while in the male rats induced evident sedative effects, as indicated by decreased locomotor activity [93]. It seems that the anxiolytic effect of *E. amoenum* is sex-dependent and probably due to phytosterol components in this plant, which compete with cholesterol absorption diminishing cholesterol and hence testosterone levels [94-96]. An experimental study also compared the effect of aqueous extract of *E. amoenum* with buspirone on the anxiety and reported that administration of *E. amoenum* (150 mg/kg) for seven days induced an anxiolytic-like effect less than buspirone, while its impact increased with chronic treatment for 14 days [97]. The results of a double-blind, randomized clinical trial showed that aqueous extract of *E. amoenum* (500 mg/day) in combination with fluoxetine (20 mg/day) for eight weeks has an anxiolytic effect, starting from the second week, without any side effects [18]. Another human study demonstrated that aqueous extract of *E. amoenum* at dose 500 mg/day for six weeks attenuated obsessive and compulsive symptoms and induced an anxiolytic effect, without any severe side effects [16]. Evidence shows that flavonoids have mild sedative and anxiolytic effects. According to the earlier studies, flavonoids and their artificial byproducts particularly attach to the central benzodiazepine receptors, inducing anxiolytic, and other benzodiazepine-like effects [98]. Therefore, it is likely that the anxiolytic effect of *E. amoenum* is due to high flavonoids content in this plant.

#### 3.2. Depression

Depressionis an affective disorder characterized by helplessness and anhedonia, resulting in enormous personal suffering and socioeconomic burden [99]. Increased oxidative stress and neuroinflammation are proposed as the principal factors in the pathogenesis of depression and anxiety [100-103]. Oxidative stress is associated with different structural and functional impairments in the brain. Evidence shows that agents with antioxidant and anti-inflammatory properties can improve depressive mood in human and animals model [104, 105]. Treatment with available conventional drugs is limited by unexpected side effects such as sedation, insomnia, tachycardia, postural hypotension, and blurred vision. Moreover, numerous patients show intolerance or refractory reactions to these drugs, which is associated with functional impairment and low quality of life [106]. Therefore, many studies have investigated alternative options such as phytomedicine as complementary remedies for treatments of depression [107]. A preliminary randomized, double-blind clinical trial, for the first time, revealed that aqueous extract of *E. amoenum* (375 mg/day, for six weeks) improved depressive symptoms, as assessed by the Hamilton Rating Scale for depression, at week four of the study, although this effect was not maintained at week six. However, this herbal treatment demonstrated no distinctive anxiolytic activity [17]. The antidepressant effect of *E. amoenum* is possibly related to its flavonoids, saponins and unsaturated sterols components, since previous studies confirmed the antidepressant activity of flavonoids which is comparable to that of fluoxetine and imipramine [89, 108, 109]. An experimental study reported that two-week oral administration of aqueous extract of *E. amoenum* (125 mg/kg) increased serotonin and dopamine levels in the cerebrospinal fluid (CSF) of reserpine-induced depression rats [110]. A recent study showed that oral administration of *E. amoenum* extract (5 mg/kg) for 15 days improved depression-like behaviors induced by manganese (Mn^2+^), including body weight gain, sucrose consumption, and immobility time in the forced swimming test. Moreover, *E. amoenum* attenuated Mn^2+^ neurotoxicity by reduction of ROS levels and lipid peroxidation as well as apoptosis in the hippocampus of Mn^2+^-treated rats. Also, *E. amoenum* extract increased catecholamine content in the hippocampus [111]. A recent human study compared the effectiveness of *E. amoenum* syrup with citalopram, an antidepressant, in the treatment of major depression. They found that eight weeks administration of citalopram or *E. amoenum* syrup reduced depression symptoms, as assessed by Hamilton depression rating scale. Interestingly, *E. amoenum* was more effective than citalopram in attenuating depressive symptoms, and its complications were also lower than citalopram at the 8^th^ week [112].

#### 3.3. Seizure

Seizure is defined as spontaneous hypersynchronous discharges of the cortical neurons due to a sudden imbalance between the excitation and inhibition, resulting in a temporary disruption of the brain function. Its clinical manifestations depend on which specific region of the *brain* is *involved* as well as the extent of brain involvement [113]. Methanolic extract of *E. amoenum* flowers at dose 6.25 mg/kg has been shown to delay the onset of the seizure, decrease the severity, and mortality of seizure-induced by picrotoxin, a GABA_A_ antagonist, in mice. Previous studies have shown that γ-linoleic acid and flavonoids have anticonvulsant effects [114-117]. Therefore, it seems that the anticonvulsant effect of *E. amoenum* is due to the presence of linoleic acid and flavonoids compounds [21, 98].

#### 3.4. Pain

Pain is an unpleasant sensation, which is linked to the activation of nociceptors by different noxious stimuli [118]. Hyperalgesia, an increased response to a noxious stimulus, is a consequence of tissue insults and is accompanied by an increased sensitivity to pain in the nervous system. Two main types of hyperalgesia, primary and secondary, are attributed to different mechanisms. Primary or peripheral hyperalgesia is induced by the release of various inflammatory mediators at the injury site, while secondary or central sensitization results from increased pain transduction or decreased inhibition in the nociceptive pathways [119]. Methanolic extract of *E. amoenum* at the dose of 10 mg/kg has been shown to decrease pain response in the late phase of the formalin test as well as in the hot-plate test in the mice. Moreover, in the hot-plate test, 45 min after injection of *E. amoenum* at dose 10 mg/kg, the peak of analgesic effect was seen. Besides, pretreatment of the animal with naloxone, opioid receptor antagonist, 5 min before administration of the extract suppressed its analgesic effect in the hot-plate and acute phase of formalin tests, suggesting that analgesic activity of *E. amoenum* is at least relatively facilitated by opioid receptors [20]. In the formalin test, the early phase is due to direct stimulation of nociceptors while the late phase is secondary to the inflammatory reaction. Therefore, it is likely that the antinociceptive effect of *E. amoenum* in the late phase of the formalin test is caused by its anti-inflammatory effects [23]. Another possible explanation for the analgesic effect of *E. amoenum* is the presence of RA and flavonoid, whose analgesic and anti-inflammatory activities have been previously reported [38, 120, 121].

#### 3.5. Ischemia

The ischemic stroke is associated with abrupt interruption of the blood flow to part or all of the brain leading to neuronal death accompanied by a permanent loss of neuronal function and long-term disability. Ischemia-induced neuronal injury is a result of a series of events including ATP loss and energy depletion, ionic imbalance, mitochondrial dysfunction, oxidative stress, and post-ischemic neuroinflammation ultimately leading to neuronal cell death [122]. In a study, the protective effect of different doses (50, 100, and 200 mg/kg, i.p) of *E. amoenum* on neurobehavioral deficits-induced by transient global cerebral ischemia for 30 min followed by 72 h reperfusion were investigated. The results showed that the extract at dose 200 mg/kg improved spontaneous activity, while it could not enhance the ability of climbing, body proprioception, and response to vibrissae touch. However, the extract at dose 200 improved inhibitory avoidance memory along with attenuation of neuronal damage in the CA1 hippocampus. Although *E. amoenum* extract reduced myeloperoxidase activity, a marker of leukocyte infiltration, it did not decrease brain edema [123]. Given that anthocyanins possess potent antioxidant and anti-inflammatory effects, it seems that the protective effect of *E. amoenum* is, at least partially, through its anthocyanins content. In an in vitro ischemia model of retina ganglion cells (RGCs) line, the ethanolic extract of *E. amoenum* at 5 μg/mL increased GSH and glutathione-S-transferase (GST) levels, decreased apoptotic cells, and maintained cell viability. In the in vivo preparation, *E. amoenum* ethanol extract at doses 200 and 400 mg/kg reduced inflammatory response induced by ischemic optic neuropathy in mice model [124].

#### 3.6. AD

AD, the most common cause of dementia, is a chronic and irreversible neurodegenerative disorder. AD is associated with gradual and permanent impairments of memory and cognitive performance. Several mechanisms have been proposed in the pathophysiology of AD including accumulation of Aβ plaques, mitochondrial dysfunction and oxidative damage, impairment of cholinergic system, neuroinflammation, and glutamate neurotoxicity [125-129]. Among them, extensive studies support the causal role of the brain mitochondrial dysfunction and oxidative damage in the initiation of AD pathology [130-132]. Medicinal plants are considered as sources of natural antioxidant components such as polyphenols, which play a role in scavenging free radicals. Therefore, the application of herbal antioxidants can protect the brain against major neurodegenerative diseases such as AD and PD [133, 134]. Sadeghi *et al*. have reported that *E. amoenum* extract at dose 50 mg/kg for 15 days improved spatial memory in the Morris water maze (MWM) task of the nucleus basalis of Meynert (NBM)-lesioned rat model of AD. Moreover, this treatment decreased ROS levels, lipid peroxidation, and reduced AChE enzyme activity accompanied by reduced degenerated cells in the hippocampus of NBM lesioned rats [19].* Since E. amoenum* is a potential source of flavonoids and RA, it seems that these constituents can protect the brain from diseases attributed to the oxidative damage, namely AD, through scavenging free radicals [6, 22]. Moreover, these contents of *E. amoenum* can decrease the activity of AChE enzyme resulting in an increase in the acetylcholine levels and improvement of memory function [33]. Table-1 summarized existing evidence on duration, form, and route of *E. amoenum* administration in neuropsychiatric disorders.

### 4. Toxicity and Side Effects

Unfortunately, there is no standard of safety and toxicological tests for most of the herbal extracts [135]. The common concept is that these products are not toxic, which is incorrect and dangerous. Pyrrolizidine alkaloids are present in a number of herbs such as *B. officinalis*, and they can induce hepatotoxicity, genotoxicity, and carcinogenicity in both animals and humans upon excessive or prolonged usage [136]. Rabbani *et al*. demonstrated that* E. amoenum* in the concentration above 50 mg/kg produces severe sedative effects [90]. Zahedi *et al*. have reported that administration of the methanolic extract of *E. amoenum* at dose 200 mg/kg for seven days increasesalanine transaminaseandaspartate aminotransferaselevels,sensitive indicators of liver damage. However, no nephrotoxicity was reported at this dose [137]. An in vitro study also found that both hydro-alcoholic and aqueous extracts of *E. amoenum,* in the concentrations of 25 mg/ml, cause DNA damage and genotoxicity [138]. Toxicity of this herb is attributed to the presence of pyrrolizidine alkaloids, namely echimidine I, echimidine isomer II, 7-angeloylretronecine III, and 7-tigloyl retronecine IV [139, 140]. However, evidence shows that its total alkaloid content is 0.01% [139].Nevertheless, Mehrabani *et al*. have reported that *E. amoenum* at dose 800 mg/kg by oral gavages for 28 days has no hepatotoxicity effect in rats [141]. Likewise, Zamansoltani *et al*. have shown that 7-14 days’ administration of aqueous extract of *E. amoenum* at doses of 100, 200 or 400 mg/kg/day reduced serum levels of alanine transaminase and alkaline phosphatase, and no sign of distinctive hepatotoxicity was observed in rats [142]. A recent *in vitro* study showed that extracts of hexane, dichloromethane, and ethyl acetate of *E. amoenum* had no cytotoxic effects on macrophage cell line at concentrations of 1-100 μg/mL; however, the concentration of 200 μg/mL decreased cell viability [23].Fortunately, no major adverse side effect has been reported for *E. amoenum* in different studies. The most common set of unwanted side effects are blurred vision, headache, dry mouth, vomiting, and constipation [17].

## Conclusion

Overall, various forms of *E. amoenum* extract has beneficial effects on neuropsychiatric disorders, and its biological activities stem from different chemical compounds such as RA, flavonoids, and anthocyanin. Given the widespread consumption of this plant in Iranian folk medicine, more studies are needed to elucidate the exact processing methods and mechanism of action of this plant in brain disorders. Furthermore, well-designated clinical studies are necessary to confirm the therapeutic or side effects of *E. amoenum* products in human.

## Acknowledgment

This paper was supported by a grant from the Neuroscience Research Center of Tabriz University of Medical Science (grant number: 61431).

## Conflict of Interest

There is no conflict of interest with this work.

**Table 1:** Current Evidence of the Effects of E. amoenum on Neuropsychiatric Disorders

**Disorders**	**Pre-clinical evidence**	**Clinical evidence**
**Depression**	Aqueous extract for 2 weeks (oral) [110] Aqueous extract for 15 days (oral) [111] Syrup for 8 weeks [112]	Aqueous extract for 6 weeks (oral) [17]
**Anxiety**	Hydroalcoholic extract, 30 min before the test (i.p.) [89] Ethanolic extract 30 min before the test (i.p.) [90] Aqueous extract for 15 or 30 days (i.p.) [91] hydroalcoholic extract 30 min before the test or for 7 days (i.p.) [92] Herbal tea for a day [93] Aqueous extract for 7 or 14 days [97]	Aqueous extract for 8 weeks (oral) [18]
**Alzheimer’s Disease/memory enhancer**	Aqueous extract for 15 days (oral) [19] Total anthocyanin extract 30 min before the induction of cerebral ischemia (i.p.) [123]	-
**Obsessive compulsive disorder**	-	Aqueous extract for 6 weeks (oral) [16]
**Seizure**	Methanolic extract 20 min before picrotoxin (i.p.) [21]	-
**Pain**	Methanolic extract 45 min before the test (i.p.) [20]	-
